# A system science perspective on burn-out: development of an expert-based causal loop diagram

**DOI:** 10.3389/fpubh.2023.1271591

**Published:** 2023-11-16

**Authors:** Lisa S. Barsties, Saskia W. van den Berg, Stephanie S. Leone, Mary Nicolaou, Sandra H. van Oostrom

**Affiliations:** ^1^National Institute for Public Health and the Environment (RIVM), Bilthoven, Netherlands; ^2^Department of Mental Health & Prevention, Trimbos Institute, Netherlands Institute of Mental Health and Addiction, Utrecht, Netherlands; ^3^Department of Public and Occupational Health, Amsterdam University Medical Centers, University of Amsterdam, Amsterdam, Netherlands; ^4^Centre for Urban Mental Health, University of Amsterdam, Amsterdam, Netherlands

**Keywords:** burn-out, societal developments, living and working conditions, complexity science, group model building, causal loop diagram, feedback loops

## Abstract

**Introduction:**

Burn-out leads to reduced worker well-being, long-term absenteeism, and high costs for employers and society. Determinants at different levels may affect burn-out in an interrelated and dynamic manner. The aim of the present study was to apply a broader systems perspective by exploring and visualizing the complex system of determinants at different levels (living conditions, working conditions, and societal developments) underlying the prevalence of burn-out in the Netherlands.

**Methods:**

During three group model building (GMB) sessions with in total eight experts on workers’ mental health, a causal loop diagram (CLD) was developed and relevant feedback loops were identified. For the selection of determinants to be included in the CLD a recently published overview of determinants on burn-out at different levels was used. Experts could also add factors that were not listed in the overview.

**Results:**

The final CLD consists of 20 factors and depicts a central position of working conditions. Societal developments (e.g., access to mental health care, size of the working population, rougher social climate, etc.) were mostly located at the outside of the CLD and barely integrated in feedback loops. Several reinforcing feedback loops resulting in an increase of the prevalence of burn-out were identified in which the factors (very) high workload, imbalance between work and private life, and insufficient recovery time play an important role. Also, several balancing loops were found that visualize the crucial role of functional support from supervisors to prevent burn-out among workers.

**Discussion:**

Applying a broader systems perspective, including determinants at different levels, offers new insights into dynamic feedback loops that contribute to the prevalence of burn-out. Supervisors, amongst others, have a considerable impact on the system underlying the high prevalence of burn-out and may therefore contribute to its prevention. Even though societal developments were less integrated in feedback loops, they might be considered drivers of existing feedback loops. The results from this study confirm that determinants at various levels underly the prevalence of burn-out. To be able to address the diversity of determinants underlying a high prevalence of burn-out, a complex system approach can be helpful.

## 1. Introduction

Prolonged work-related stress affects a high percentage of the working population and organizations worldwide (1–3). In Europe, on average around 10% of the workforce feels burned-out (4). In the Netherlands, the share of workers with burn-out complaints was 17% (1.3 million) in 2021 (5), while in 2010, it was 13% (6), indicating an increase over the past years. Worldwide, the concept of burn-out and its definition are cause for discussion (7). A frequently used definition of burn-out – but certainly not the only one – describes it as a syndrome characterized by three main dimensions: emotional exhaustion, increased mental distance from or feelings of negativism/cynicism related to one’s job, and reduced professional efficacy (8, 9). Also, in some countries (e.g., the Netherlands) burn-out is a recognized illness in occupational health, whereas other countries (e.g., the United States) treat it as a non-medical syndrome, having serious consequences for insurance (10). Negative consequences of burn-out include reduced worker well-being (11), long-term absenteeism (11), and high costs for employers and society (3).

Determinants on different levels - such as the, individual, organizational, and societal level - underlie the development of burn-out (12). Research has shown that individual factors, such as lifestyle factors (i.e., sleep and physical exercise) (12–15), demographic factors (i.e., age and sex) (8, 16, 17), and coping strategies (8, 12, 18, 19), play a role in the development of burn-out. These factors are relevant as they explain inter-worker differences in the development of burn-out within an organization. Organizational determinants of burn-out, relating to aspects of the workplace, also have been frequently studied. Examples of organizational determinants of burn-out are high emotional job demands resulting from interactions with clients (8, 20, 21), job insecurity (22), low social support of colleagues and supervisors (8, 18, 23), a high workload and low autonomy (8, 18, 24), and an unhealthy organizational culture (25). Research on macro-level determinants of burn-out, such as societal developments (i.e., declining working age population, digitalization/technologization, and economic demands, such as the 24/7 mentality), is scarce but may be important as they affect organizations and workers’ psychosocial workload considerably (26, 27).

Individual, organizational, and societal determinants most likely affect burn-out in an interrelated and dynamic manner. Yet, the majority of epidemiological research focuses on a specific relationship only, aiming to find a linear relationship between determinant and outcome (28) and thereby disregarding the complexity, non-linearity, and interrelatedness of determinants. To acknowledge these aspects, examining burn-out from a complexity science perspective is necessary. This perspective facilitates the identification of interactions among various elements within a system (29). A recent study (30) developed a system dynamics model (i.e., a model describing the structure and behavior of a system) of burn-out, including individual and organizational determinants. It explicitly accounted for the complexity of the interrelatedness of various determinants and their impact on burn-out. This study was an important contribution to the understanding of the development of burn-out at the individual-level. Yet, they argued that more aspects of the context in which individuals live and work should be taken into consideration. Therefore, the aim of the present study was to explore and visualize the complex system of living and working conditions as well as societal developments underlying the high prevalence of burn-out in the Netherlands. To do so, a qualitative causal loop diagram (CLD) was developed during group model building (GMB) sessions with experts.

## 2. Materials and methods

### 2.1. Design

GMB is a participatory systems modelling approach to involve stakeholders in the development process of a causal map (CLD) or a computer simulation model (31). During GMB sessions, insights concerning a problem are shared, factors are selected, and their relations with each other are visualized (32, 33). A CLD is a qualitative conceptual model that visually represents key factors underlying a complex problem and their interconnectedness (i.e., their causal relationships) (34). Connections between factors are visualized by arrows, that are either solid (i.e., an increase in the causal factor leads to an increase in the effect factor) or dashed (i.e., an increase in the causal factor leads to a decrease in the effect factor). Also, CLDs are a powerful tool to identify the underlying feedback loops (i.e., causal chains of factors that start and end at the same factor) as well as leverage points in a system (35). Feedback loops can either be reinforcing or balancing. Reinforcing feedback loops strengthen or exacerbate existing movements in a system. Balancing feedback loops stop further increase in a given direction, regulating the system and thereby bringing it to a desired state (36).

### 2.2. Participant recruitment process

As we aimed to develop a conceptual model visualizing key factors underlying the high prevalence of burn-out as well as their interconnectedness, that is supported by knowledge on empirical research literature, we included experts as participants. The GMB sessions were held with academic experts on workers’ mental health working for knowledge institutes, universities, and occupational health services. We aimed to include Dutch speaking experts with at least 4 years working experience in the field of workers’ mental health. Also, the experts were selected to represent different relevant organizations. We invited 12 experts for the GMB sessions by sending them an e-mail with detailed information on our study. A total of eight experts agreed to participate; at each GMB session seven experts were able to be present.

All experts provided written informed consent. The Centre for Clinical Expertise of the National Institute for Public Health and the Environment concluded that this study did not fall under the Dutch Medical Research Involving Human Subjects Act (WMO). Therefore, a formal ethical approval was deemed not to be necessary. The experts received a gift voucher for their participation.

### 2.3. Procedure

**Figure 1:**
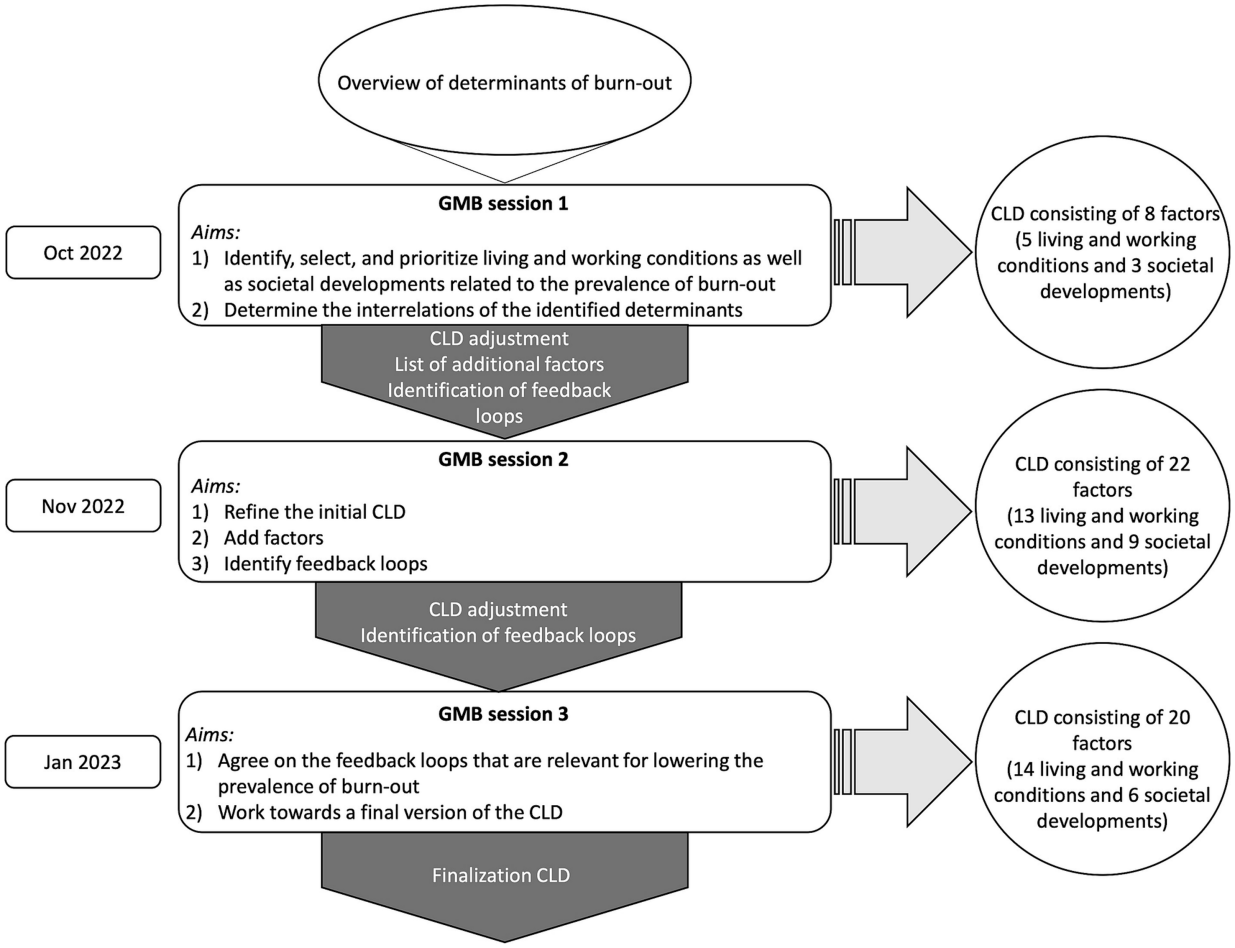
Schematic overview of the 3 GMB sessions, their aims, and results.

Figure 1 depicts a schematic overview of the study procedure. To increase transparency, replication, and the transmission of good practices (33), there are GMB scripts available that are freely accessible at https://en.wikibooks.org/wiki/Scriptapedia. These scripts were used to prepare and guide the GMB sessions of this study and are described in more detail below.

We used a recently published overview of determinants significant related to burn-out (37) for the selection of factors during the GMB sessions. In this overview, 61 relevant determinants based on existing literature are divided into three levels of influences according to The Dahlgren-Whitehead rainbow model (38). The first level describes individual and lifestyle factors (n = 11) divided into 3 themes for example coping skills and demography. The second level describes living and working factors (n = 31) divided into 11 themes for example social support and organizational culture. The third level includes more general societal conditions (*n* = 19) divided into 5 themes for example economy and politics. Experts could also add factors to the CLD that were not listed in the overview, if they were considered relevant. Working with a pre-existing list of evidence-based determinants from which experts can select and possibly add factors to, offers an successful starting point for the development of a CLD (39). Moreover, presenting a broad spectrum of determinants at different levels and themes can motivate experts to think broader than their own scientific expertise. Finally, there is evidence that the application of a theoretical model in the creation of a CLD results in a more robust outcome (40). As we aimed to visualize living and working conditions as well as societal developments underlying the high prevalence of burn-out, we did not primarily focus on individual factors and excluded them from the selection process. We did not aim to include as many factors as possible or even all factors listed in the overview, but rather to make a selection of factors that are considered the most relevant by our group of experts. For an overview of the living and working conditions and societal developments that are listed in the overview, see Supplementary File 1.

The GMB sessions were held between October 2022 and January 2023. The first two GMB sessions were physical meetings, the third session was organized online. The first workshop lasted 3.5 h, the second workshop lasted 2 hours, and the third workshop lasted 1.5 h. The workshops were led by members of the research team, consisting of five researchers, who took up the roles of facilitator, wall builder (visually structures and organizes selected factors into thematic clusters, see script wall builder), and note-taker. The workshops were documented with audio recordings, written notes, and photographs.

#### 2.3.1. GMB session 1

The aims of the first session were to (1) identify, select, and prioritize living and working conditions as well as societal developments related to the prevalence of burn-out, and (2) to determine the interrelations of the identified determinants. At the beginning of the first session, we introduced the basics of complex systems sciences and the topic of our research, to develop a shared understanding of its complexity. The experts were asked to select living and working conditions and societal developments from the recently published overview of determinants. They could also add factors that were not in the overview, if they thought they were relevant. To guarantee that factors from both levels were selected, this exercise was done in two separate rounds: first, the experts could select factors from the living and working conditions, then, they could select societal developments. To do so, the experts received empty graphs (with time on the X-axis) and were asked to fill in a selected factor on the Y-axis as well as to draw its future development if (a) current trends continued and (b) if intervention(s) would be implemented (Script: Graphs over time). The aim of the graphs was to motivate the experts to think of factors that can change over time. This can help participants to think of factors as dynamic and non-static parts of a system as opposed to elements of linear relationship between determinant and outcome. They could individually fill in as many graphs as they wanted and were asked to sort them by perceived importance. Subsequently, the experts presented their top two factors for each level to the group. This resulted in 16 factors relating to living and working conditions and 14 factors relating to societal developments.

To prioritize the selected factors, each participant received ten stickers (five for each level) to place on the graphs that they perceived to be most relevant. Again, living and working conditions and societal developments were prioritized in two separate rounds (Script: Dots). Multiple dots could be placed on the same graph. The higher the number of dots on a graph, the more relevant it was considered. This exercise was done individually and the result was discussed plenary. Factors with the highest number of stickers were used to build a first version of the CLD. The prioritization resulted in eight factors to be included in the CLD (5 living and working conditions (3–5 stickers) and 3 societal developments (4 stickers)). Finally, the group, as a whole, was asked to connect the factors with each other by cause and effect relationships (Script: Creating causal loop diagram from variable list). This was done until all 8 factors were integrated in a first version of the CLD.

Following the first GMB session, the researchers adjusted the CLD (see Analysis). They also prepared a list of additional factors between the first two sessions, from which the experts could select factors to be added to the CLD during the second session. This list included 10 factors (see Supplementary File 1) that were prioritized during the first session, but did not receive enough stickers to be included in the CLD (5 living and working conditions (2 stickers) and 5 societal developments (3 stickers)). Of these, 3 living and working conditions were excluded from the list because they represented a conceptual model instead of an individual factor (i.e., effort-reward imbalance model) or because they overlapped with other factors (i.e., poor housing, and access to help). Finally, 3 factors were added to the list that were not prioritized high enough during the first session but were considered to be too relevant (based on existing scientific evidence) by the research team to not be included (i.e., autonomy, coworker support, and job security).

#### 2.3.2. GMB session 2

The aim of the second session was to refine the initial CLD and to identify feedback loops, thereby visualizing how interrelated determinants impact burn-out and *vice-versa*. At the beginning of the second session, changes made to the CLD by the researchers were explained and discussed with the participating experts. The experts were then invited to expand the CLD by adding factors from the predefined list of 10 factors or by adding factors they considered relevant but were not included in the first version of the CLD or the predefined list. In total, the experts added 14 factors to the CLD (see Supplementary File 1). To introduce the concept of feedback loops, 5 loops that were identified by the researchers were presented to the experts. Experts were asked to identify more feedback loops in the adapted CLD. Adding factors and finding feedback loops was first done in pairs and then presented to the group.

Following the second GMB session, the researchers adjusted the CLD again (see Analysis). Prior to the third GMB session, the experts were asked by email to select the feedback loops they considered most relevant for lowering the prevalence of burn-out (maximal three) out of six loops that were identified by the researchers. Experts could also identify additional feedback loops or describe variations of the feedback loops that were identified by the researchers. Five of the six experts sent their choices, including the reason for their choice by email before the third session.

#### 2.3.3. GMB session 3

The aim of the third session was to agree on the feedback loops that are relevant for lowering the prevalence of burn-out and work towards a final version of the CLD. At the beginning of the third session, the experts could react to changes made to the CLD by the researchers. The loops that were selected after session 2 were presented and discussed plenary. It was also decided to split the factor ‘disbalance work-private life’ into 2 factors (i.e., ‘private life interference with work’ and ‘work interference with private life’).

### 2.4. Analysis

After the first session, the CLD was transferred to the modelling software Vensim PLE (version 9.3.4). Vensim was also used to identify feedback loops and analyse which factors are most embedded in causal mechanisms related to the outcome ‘prevalence of burn-out’ (Causes Tree Diagram). To improve the CLD’s visual quality, it was ultimately transferred to the modelling software Kumu. Between the sessions, the team of researchers adjusted the CLD by improving factor names, removing illogical connections, and adding missing connections. These adjustments were done based on insights from existing literature and expert knowledge of the authors. Supplementary File 1 provides an overview of the iterative process of building the CLD (i.e., the factors the experts could choose from, the prioritization of factors, removed factors, and adjusted factors). The experts were sent a report of the content and output of the first and second GMB session in preparation of the second and third session, respectively. The reports also summarized the adjustments made to the CLD by the researchers. This enabled the experts to follow, understand, and if necessary react to the adjustments.

## 3. Results

### 3.1. Causal loop diagram

**Figure 2:**
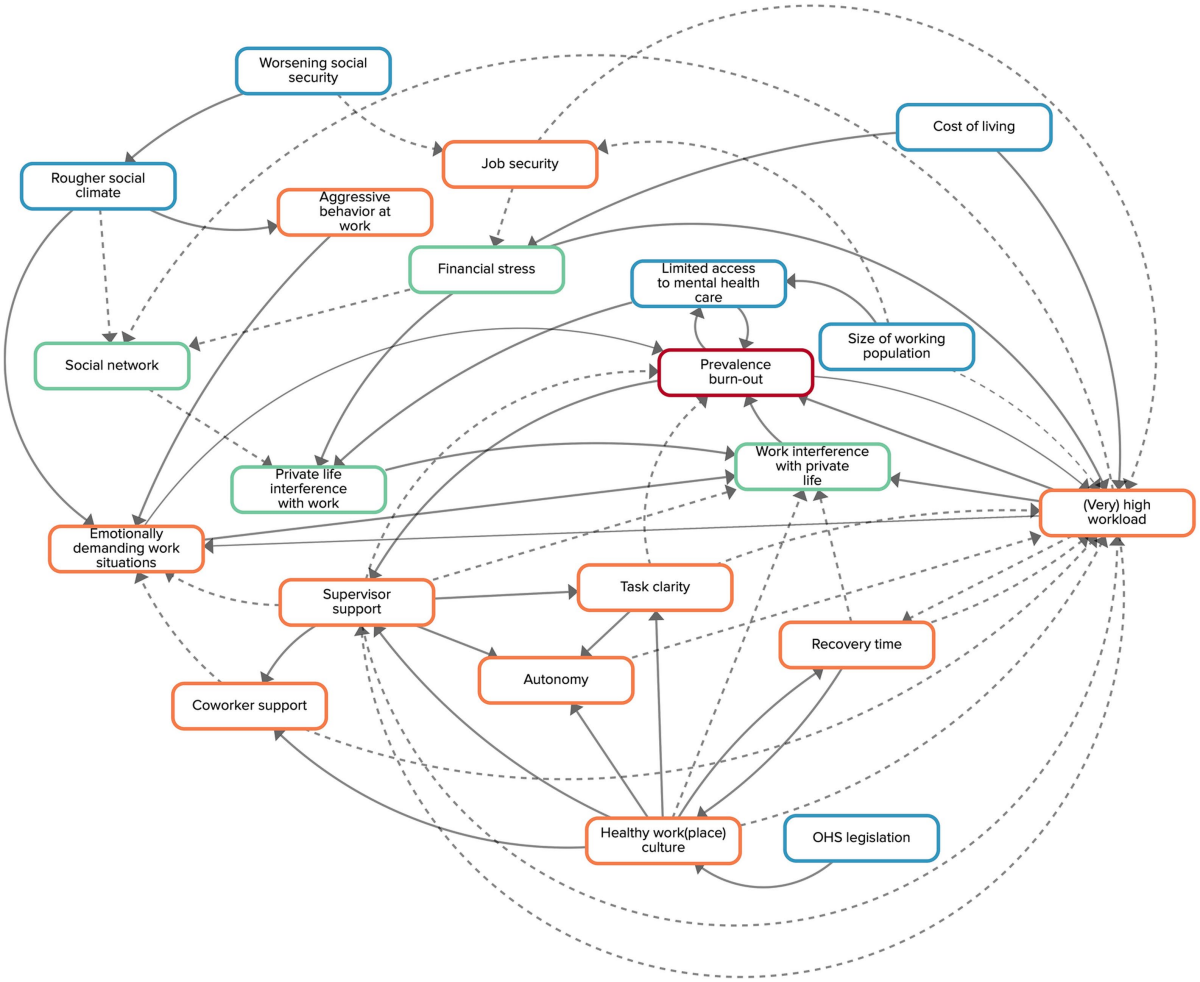
Causal loop diagram made by the experts. Orange factors relate to working conditions, green factors to living conditions, and blue factors to societal developments. Factors are connected by arrows, indicating the direction of the relationship. Solid arrows indicate that both factors move in the same direction, dashed arrows indicate a counteracting relationship.

There are 20 factors in the final CLD (Table 1). Figure 2 displays the CLD, illustrating the relationships between all factors. The 10 orange factors relate to working conditions, the 4 green factors to living conditions, and the 6 blue factors to societal developments. Five working conditions [i.e., ‘working (extra) hard’, ‘work interference with private life’, ‘supervisor support’, ‘healthy work(place) culture’, and ‘emotionally demanding work situations’] show a high number of in- and outgoing arrows. Other factors, especially the ones relating to societal developments, have less or no ingoing arrows. The CLD shows the dynamic nature of the system which is visualized in a high number of feedback loops. In the following, we will only present and describe the feedback loops that were thoroughly discussed and considered to be relevant by the experts during the GMB sessions. The description of the feedback loops reflects the discussions of the experts during the GMB sessions. Not all factors that appear in the text are part of the feedback loops; some of them were simply named by the experts to explain the mechanisms that are represented by the loops. The experts identified 3 reinforcing feedback loops, meaning that they lead to an increase of the prevalence of burn-out, and 2 balancing loops, which means they lead to a lower prevalence of burn-out.

**Table 1:** Overview of factors in the final CLD.

Level	Factor	Definition
Living conditions	Social network	A person’s social network of friends, relatives, and acquaintances.
	Financial stress	Stress arising when perceiving that one’s income cannot fulfill financial obligations.
	Private life interference with work^a^	Work-life imbalance due to private life (e.g., care responsibilities) interfering with work.
	Work interference with private life^a^	Work-life imbalance due to work interfering with private life.
Working conditions	Job security	The probability of keeping one’s job and in case of losing it finding a new one.
	Aggressive behavior at work	Aggressive behavior in the workplace by, for example, clients, students, patients, etc.
	Functional supervisor support	Receiving functional support from one’s supervisor.
	Task clarity^a^	Having a clear understanding of one’s tasks, responsibilities, and processes at work.
	Autonomy	Being able to decide how to execute one’s work.
	Recovery time^a^	The amount of time that is granted to recover from a demanding task at work.
	Healthy work(place) culture	The organization’s degree of commitment in supporting prevention of stress and its openness to discussing mental health with employees and experts, combined with workers’ acceptance of existing (written and unwritten) rules and regulations directed at a healthy work(place) culture.
	(Very) high workload	A large volume of work to be handled by a worker.
	Emotionally demanding work situations	Emotional strain caused by emotionally difficult experiences in the workplace.
	Co-worker support	Receiving emotional and functional support from colleagues.
Societal developments	Occupational health and safety (OHS) legislation	According to the OHS legislation employers have the responsibility to minimize/prevent work-related psychosocial stress. They also are required to have an overview of possible psychosocial risk factors and implement and evaluate existing preventive measures.
	Size of working population	–
	Limited access to mental health care^a^	Access to mental health care is getting more complicated due to rules of the healthcare system as well as due to long waiting lists.
	Cost of living^a^	–
	Worsening social security^a^	–
	Rougher social climate^a^	People treating each other less kindly and respectful.

a Factors that were not listed in the overview of determinants of burn-out.

#### 3.1.1. Reinforcing feedback loops

##### 3.1.1.1. High workload

**Figure 3:**
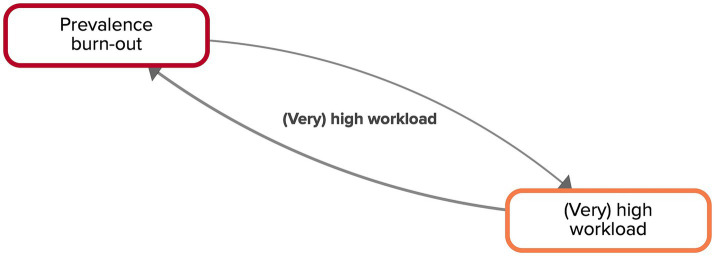
Feedback loop ‘(very) high workload’ extracted from the CLD made by the experts. Factors are connected by arrows, indicating the direction of the relationship. Solid arrows indicate that both factors move in the same direction.

Having a (very) high workload is one of the key factors in the CLD and is directly related to the prevalence of burn-out and *vice-versa*, representing a reinforcing loop (Figure 3). According to the experts, when there are more workers who have a (very) high workload, the prevalence of burn-out will rise. A higher number of workers that are unable to work, or that work fewer hours, due to burn-out symptoms, in turn, will result in a higher workload amongst their colleagues, who have to take on the tasks of their absent colleagues. This will lead to an even higher prevalence of burn-out.

##### 3.1.1.2. Imbalance between work and private life

**Figure 4:**
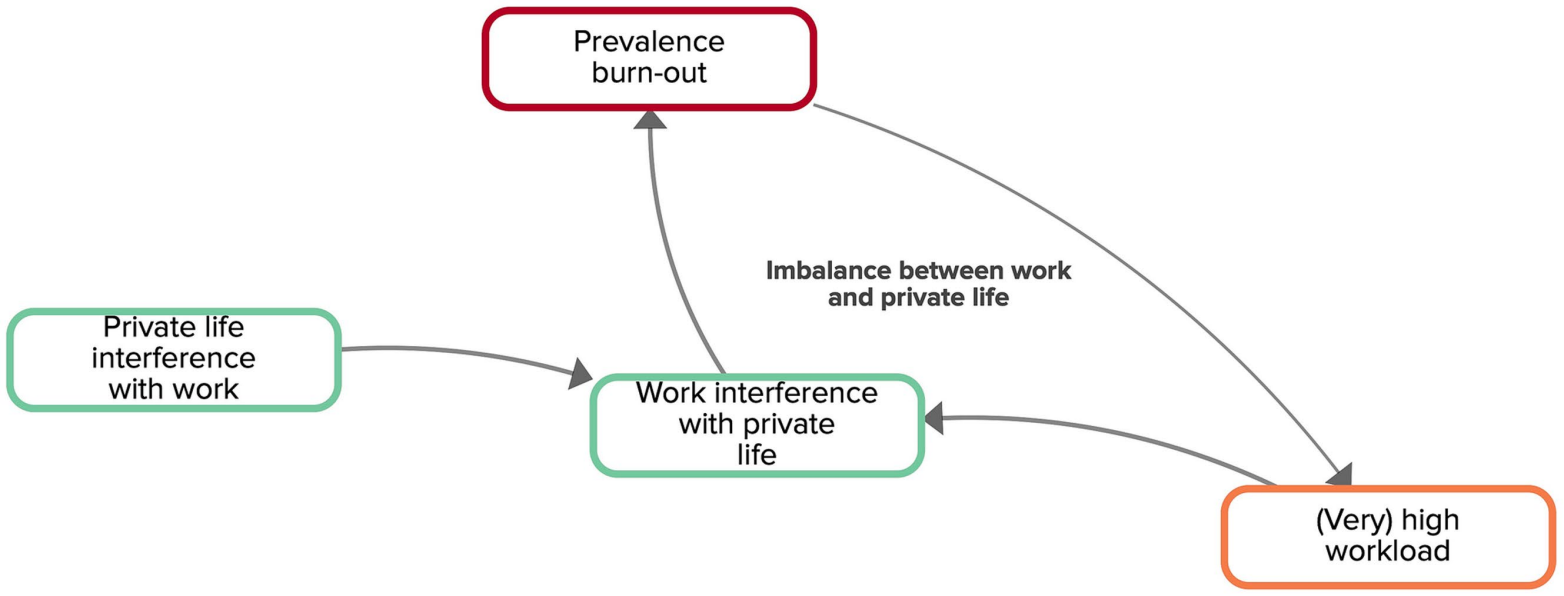
Feedback loop ‘imbalance between work and private life’ extracted from the CLD made by the experts. Factors are connected by arrows, indicating the direction of the relationship. Solid arrows indicate that both factors move in the same direction.

All experts acknowledged the important contribution of an imbalance between work and private life to the onset of burn-out. Workers that have a high workload, might experience that their work interferes with their private life. Long-term work interference with private life may lead to a higher prevalence of burn-out (Figure 4). The experts also discussed the role of private life interfering with work, for instance, when workers provide informal care to family members (including children living at home) or relatives. As there is an increased demand for informal care due to population ageing and cutbacks in residential care, more people have to combine paid work and informal care at a certain point in their working career. This can negatively affect various domains of life, such as one’s family, mental health, or social network. When informal care activities take place during work time, they could interfere with work if work cannot be done at a different time. Sometimes, informal caregivers have the flexibility to catch up on work during evening/night hours, yet, this leaves little time to recover. Often, the strict separation between time at work and private time becomes fuzzy and private life-work interference and work-private life interference are sustained during a long period of time.

##### 3.1.1.3. Insufficient recovery time

**Figure 5:**
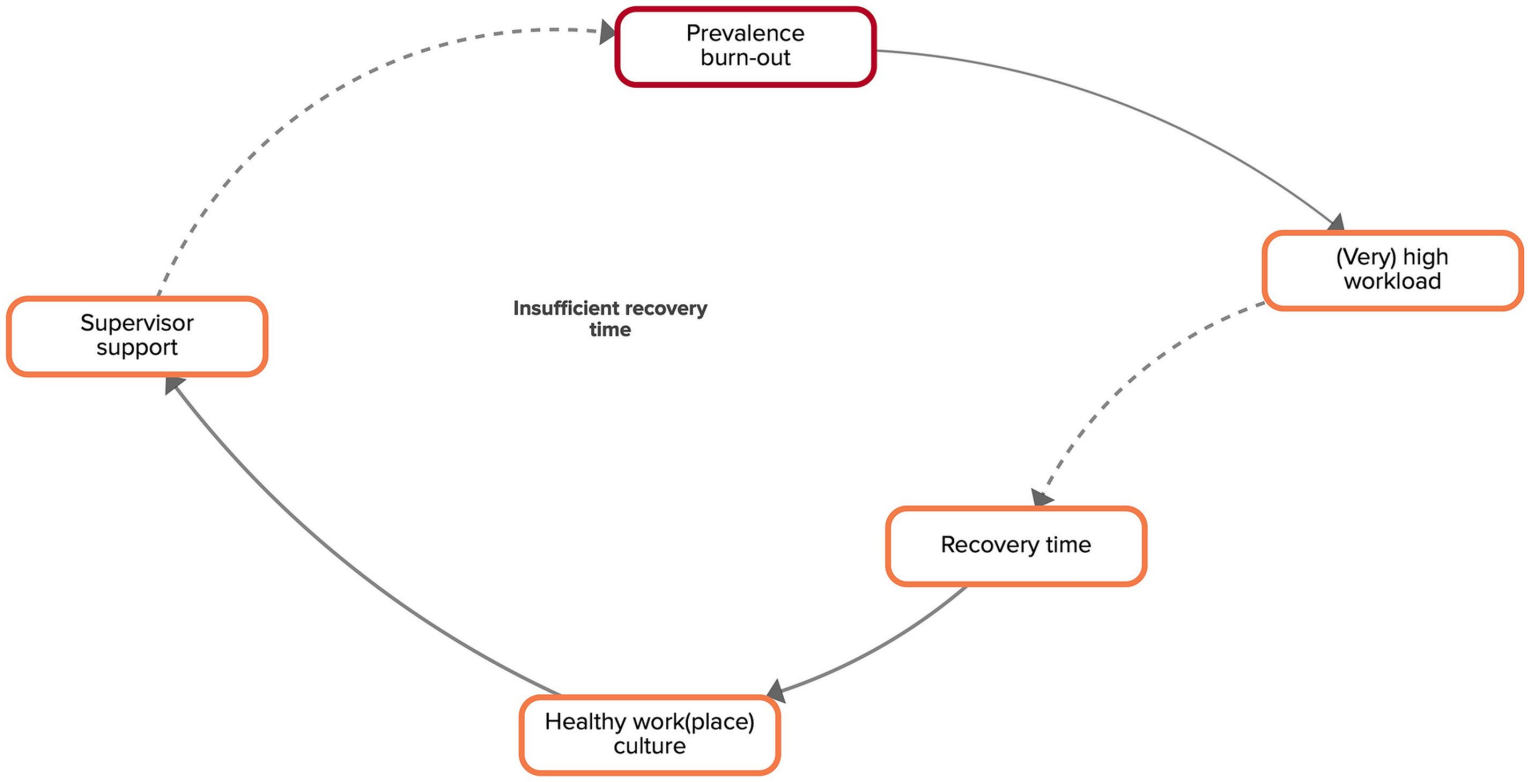
Feedback loop ‘insufficient recovery time’ extracted from the CLD made by the experts. Factors are connected by arrows, indicating the direction of the relationship. Solid arrows indicate that both factors move in the same direction, dashed arrows indicate a counteracting relationship.

The need for recovery was described by the experts to be essential for work-related health. They argued that organizations in many sectors do not offer the necessary circumstances (i.e., schedules including breaks, a place to rest, etc.) for workers to recover. Another reason for a structural lack of recovery time for workers that was discussed is that many sectors do not have clear guidelines with regard to the frequency and duration of breaks. As described above, if one or several workers in an organization are struggling with burn-out, their colleagues have a higher workload. According to the experts, this may negatively affect the time workers can take to recover as they do not have enough time to complete their (additional) tasks. Not receiving enough time and possibilities to recover during working hours can lead to feelings of exhaustion. If recovery time for workers decreases, the workplace culture will be less healthy. An unhealthy workplace culture leads to workers receiving less functional support by their supervisors, which in turn will lead to a higher prevalence of burn-out. This reinforcing feedback loop is visualized in Figure 5.

#### 3.1.2. Balancing feedback loops

##### 3.1.2.1. Role of the supervisor

All experts acknowledged the important role of the supervisor in the prevention of burn-out in an organization. Support at work – especially from supervisors – was argued to be essential for the mental health of workers. The experts argued that currently many supervisors are not well equipped to offer adequate support to their colleagues who are dealing with a high workload and/or emotional demanding situations at work. Adequate functional support of the supervisor might reduce burn-out through different mechanisms, of which two will be described in more detail.

**Figure 6:**
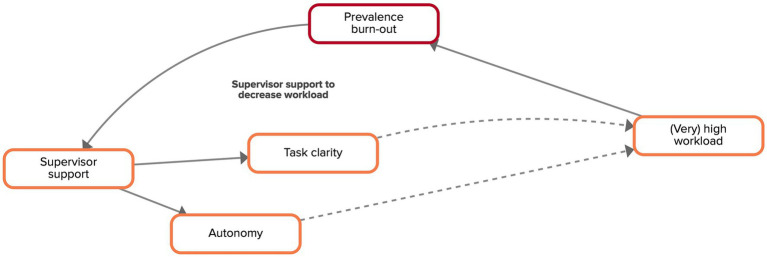
Feedback loop ‘supervisor support to decrease workload’ extracted from the CLD made by the experts. Factors are connected by arrows, indicating the direction of the relationship. Solid arrows indicate that both factors move in the same direction, dashed arrows indicate a counteracting relationship.

The experts argued that a high prevalence of burn-out could increase supervisors’ awareness of the necessity to provide support to their workers (Figure 6). This mechanism was explained by increased levels of awareness of the severity and consequences of burn-out when one or more workers in an organization are sick listed because of a burn-out. Workers who receive functional support of their supervisor will have more clarity about which tasks they have to perform as well as what these tasks entail, and they will be able to work more autonomously, which will influence their work load.

**Figure 7:**
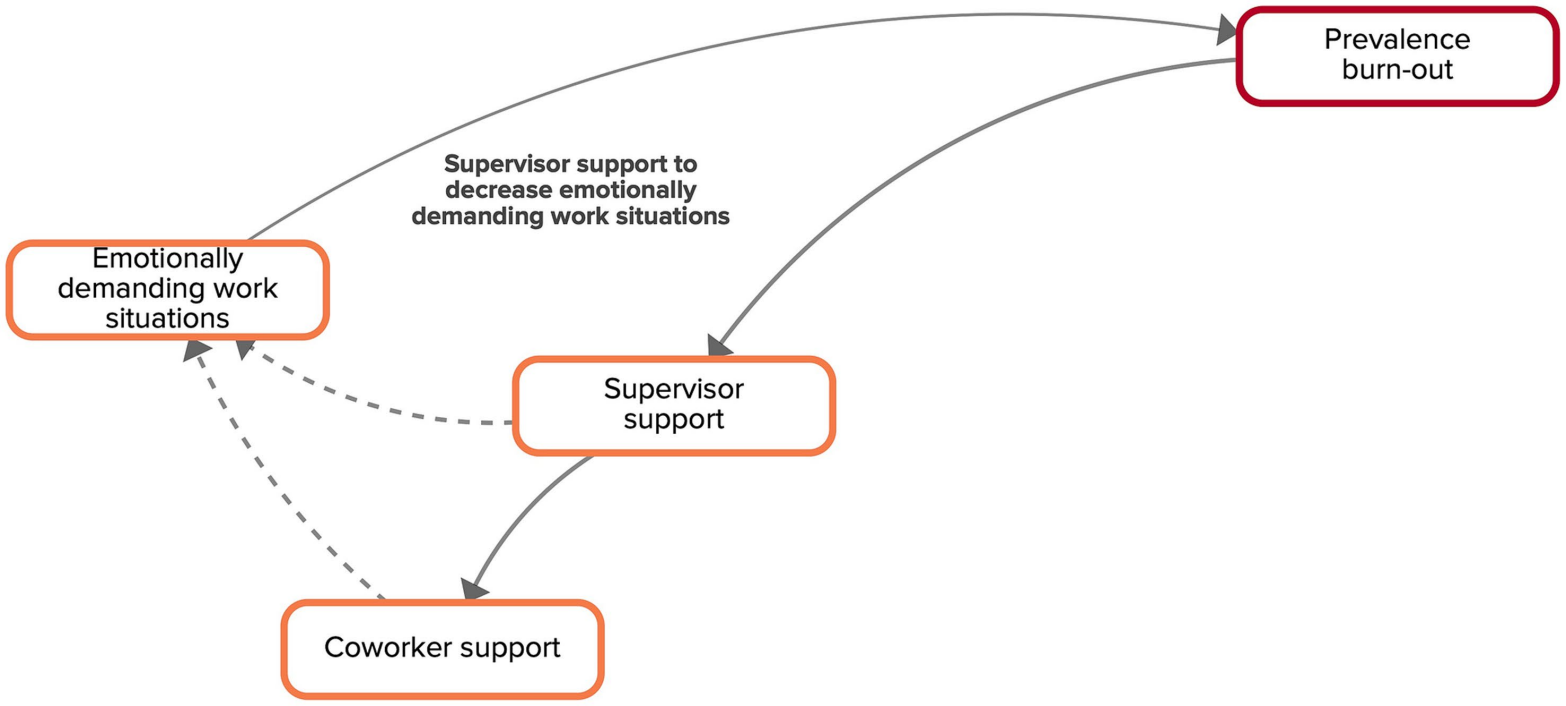
Feedback loop ‘supervisor support to decrease emotionally demanding work situations’ extracted from the CLD made by the experts. Factors are connected by arrows, indicating the direction of the relationship. Solid arrows indicate that both factors move in the same direction, dashed arrows indicate a counteracting relationship.

Functional supervisor support is also available to coworkers, creating an atmosphere in which colleagues are enabled and motivated to help each other (Figure 7). More support, both from coworkers and supervisors, can decrease the impact of emotionally demanding work situations as workers feel that they do not have to deal with them on their own.

The experts did not discuss feedback loops that include societal developments. Moreover, of all feedback loops that can be found in the CLD, only one does include a factor on the societal level (i.e., access to mental health care). Access to mental health care is directly related to the prevalence of burn-out and *vice-versa*, representing a reinforcing loop. With other words, a higher prevalence of burn-out implies that there is a bigger demand for mental health care, which ultimately might negatively affect its access. Limited access to mental health care might increase the prevalence of burn-out as people in need of support will have to wait for it, potentially worsening their burn-out symptoms.

## 4. Discussion

This study presents an expert-based CLD, which visualizes the complex system of living conditions, working conditions, and societal developments underlying the high prevalence of burn-out. Several reinforcing feedback loops resulting in an increase of the prevalence of burn-out were identified in which the factors (very) high workload, imbalance between work and private life, and insufficient recovery time play an important role. Also, various balancing loops were found that visualize the crucial role of functional support from supervisors to prevent burn-out among workers.

To our knowledge, this is the first study that aimed to delineate a complex systems model of the broader societal context and living and working conditions in relation to the prevalence of burn-out. Two previous studies developed systems models on burn-out, yet, they focus on individual and workplace determinants of burn-out complaints or workplace wellbeing (30, 39). Traditional theories on burn-out, such as for instance the job-demands resources (JD-R) model (41), also primarily focus on personal determinants and working conditions and how these affect the development of burn-out complaints in individual workers. Our CLD complements these existing models and theories, by adding a broader, dynamic perspective of the system underlying the prevalence of burn-out. In the following, we will highlight this dynamics by discussing some of the feedback loops of our CLD in light of existing literature and system models.

First, the role of functional support of supervisors for the prevention of burn-out was discussed by the experts and visualized in two balancing feedback loops. Supervisor support does not (explicitly) appear in the systems models by Veldhuis et al. and Niks et al. They do include broader workplace factors like job resources and positive work experiences (39). Yet, our CLD shows that, according to the experts, supervisor support affects some of the factors considered to be job resources (e.g., autonomy) or positive work experiences (e.g., receiving positive feedback). That supervisors play a key role in preventing burn-out is supported by a systematic review and meta-analysis that concludes that a lack of workplace support leads to burn-out symptoms (12, 23). Our CLD adds to this that workers that receive support from their supervisor experience more task clarity, autonomy, and coworker support, which ultimately may decrease the prevalence of burn-out. That supervisors are able to affect a wide range of factors that are important in the prevention of burn-out is supported by a study that found that management support is significantly associated with lower levels of emotional exhaustion and depersonalization (42). Together, these results illustrate that supervisors can have a considerable impact on the system underlying the prevalence of burn-out.

In addition, the CLD displays multiple feedback loops that include factors relating to working conditions. This emphasizes the crucial role of a healthy working environment in the prevention of burn-out. ‘(Very) high workload’ is the factor that is the most connected with other factors in the CLD. The role of a (very) high workload in the development of burn-out is also at the core of several work-stress models, such as the JD-R model (41), in which the importance of a balance between job demands and resources for individual workers is emphasized. Our CLD adds to this that the degree to which workers have to deal with a (very) high workload is not only influenced by working conditions, but also by societal developments (i.e., cost of living) and workers’ living conditions (i.e., financial stress). While no societal developments and living conditions are part of any feedback loops on the factor ‘(very) high workload’, they influence these loops from the outside by directly affecting how much individuals have to work. Societal developments and living conditions that contribute to a (very) high workload, such as cost of living and financial stress, should therefore be considered when tackling burn-out. Yet, as there is a paucity in research on societal developments that could influence the prevalence of burn-out (i.e., declining working age population, digitalization/technologization, and economic demands, such as the 24/7 mentality), future research should test how these developments affect the prevalence of burn-out, possibly through factors such as high workload.

Third, the dynamic interaction between job demands, recovery time, and burn-out is integrated in the ‘insufficient recovery time’ feedback loop. Existing research accentuates that recovery time plays a crucial role for reducing work stress (43). Also, the effort-recovery model (44) puts that in order to prevent an ongoing deterioration of well-being and performance, recovery time during working hours as well as outside of working hours is necessary. By taking a broader view on recovery time, our CLD elucidates that organizational level recovery time (i.e., circumstances that facilitate workers to recover, such as schedules including breaks, a place to rest, etc., and clear guidelines with regard to the frequency and duration of breaks) - as opposed to individual level recovery time - is relevant as it affects broader social aspects of the workplace, such as workplace culture or supervisor support, which themselves play a relevant role for the prevention of burn-out.

Finally, the reinforcing feedback loop between the prevalence of burn-out, working (extra) hard, and work interference with private life brings forward the importance of balance – between work demands, work resources, private life demands, and private life resources. This is in line with the JD-R model as well as the systems model by Niks et al. (39). Yet, by approaching the role of balance from a complexity science perspective, it becomes evident that it is harder for individuals to reach a balance between work and life when the societal prevalence of burn-out is high. That is, when the prevalence of burn-out in a country is high, individual workers will be affected by it – for instance as they have to take on tasks of colleagues who are struggling with burn-out symptoms - and will therefore have difficulties achieving work-life balance themselves.

None of the feedback loops that were discussed by the experts includes a societal development even though several societal developments have been added to the CLD. Yet, it seems plausible that societal developments are less integrated in feedback loops as they are only seldom influenced by factors on lower levels (i.e., living and working conditions). The fact that societal developments are less integrated in feedback loops does not mean they do not influence the causal mechanisms around the prevalence of burn-out. For instance, the factors ‘cost of living’ and ‘size of the working population’ influence the degree to which workers have a (very) high workload. They thereby accelerate/decelerate the 5 feedback loops in which the factor ‘(very) high workload’ is integrated. The same applies to the factor ‘rougher social climate’ that is affecting the amount of emotionally demanding work situations at work and thereby the feedback loop ‘supervisor support to decrease emotionally demanding work situations’. We therefore argue that societal developments should not be ignored as they seem to have a role in the system underlying a high prevalence of burn-out. They might be considered drivers of its causal mechanisms instead of an integrated part of them.

### 4.1. Strengths and limitations

A strength of the present study is that almost all of the selected determinants in the CLD were evidence-based. We thereby had an exhaustive list of determinants of burn-out on different levels that the experts could choose from. We believe that this increased the relevance of the factors in the final CLD.

Nearly all of the involved experts indicated that they had not worked with systems models before. They expressed that applying a systems perspective invited them to think differently about the high prevalence of burn-out. The experts indicated, for instance, that before the GMB sessions, they were neither aware of the high number of determinants of burn-out nor of their interrelatedness. This did not only enrich the present study, but might also motivate a larger group of researchers to address and study the complexity of the phenomenon of burn-out. A review of the evidence of the effectiveness of GMB (45) confirms these benefits by providing evidence that supports the efficacy of GMB (e.g., with regards to insight, communication quality, or persuasion).

Several limitations of the present study merit discussion. First, we developed a CLD together with academic experts on workers’ mental health. This might imply that knowledge that is less covered by existing research is less well represented in our CLD. Developing systems models with workers, employers, and occupational health professionals, or a different group of academic experts could help to develop additional relevant insights with regards to the position and role of societal developments in the system underlying a high prevalence of burn-out.

Second, although we did not aim to include the aspect of time in the CLD, this can be considered a limitation. That is, some causal relations may be delayed and thereby affect the system’s behavior. For example, workers who have to deal with a (very) high workload might experience that their work interferes with their private life without developing burn-out symptoms. Yet, when workers struggle with a (very) high workload for a longer period of time, they might develop symptoms of burn-out which ultimately increases the prevalence of burn-out. When developing quantitative system dynamics models in the field of burn-out, the aspect of time should be included.

Third, our systems model does not differentiate between sectors. Yet, it is plausible that factors and feedback loops function differently for different sectors. For instance, as emotional demanding work situations apply primarily to professionals who are directly involved with people (i.e., health care professionals, teachers, social workers, police (wo)men, etc.), the loop including this factor will be less relevant for workers in more administrative jobs. Therefore, future studies should develop systems models that are specified for certain sectors/types of organizations.

### 4.2. Implications for future research

To better understand how factors on different levels interrelate with each other, it would be worthwhile to integrate our CLD with existing systems models on burn-out that include factors on the individual level (30, 39). This could also help to assess the role societal developments play in feedback mechanisms between living conditions, working conditions, and individual characteristics. Also, perspectives of different stakeholders, such as professionals working in the field of work-related health (i.e., occupational physicians), but also the perspective of workers and employers should be assessed to improve the systemic perspective on the prevalence of burn-out.

The feedback loops that were identified in this study can help to find leverage points to adapt the system towards a lower prevalence of burn-out. For instance, ways to improve supervisors’ support to their colleagues should be identified and possibilities to provide sufficient recovery time should be embedded in organizational policies. That moving away from individual-level interventions to tackle burn-out is necessary has been highlighted by a recent systematic overview by Aust and colleagues (46). They found that organizational-level interventions show a moderate to strong effectiveness, suggesting that not only the work environment but also workers’ health can be improved by interventions implemented at the organizational level. Subsequently, as the system model that was developed in this study is a theoretical and abstract representation of reality, concrete actions to intervene in the system should be identified and developed together with relevant actors.

## 5. Conclusion

This study allowed us to visualize the complexity of the system underlying a high prevalence of burn-out as well as the interrelatedness of relevant factors at various levels. Applying a broader, multilevel perspective offers new insights in the currently high prevalence of burn-out in many Western countries and how to address it. More specifically, workers’ (very) high workload, their imbalance between work and private life and insufficient time to recover play an important role in exacerbating the ongoing increase in the prevalence of burn-out. On the other hand, functional support from supervisors, for instance, can regulate the system by lowering the prevalence of burn-out. We argue that interventions aimed at addressing a high prevalence of burn-out should tackle various interrelated factors, including living and working conditions as well as societal developments. Moreover, this study demonstrates the value of applying a systems perspective in burn-out research. Acknowledging and addressing the complexity underlying a high prevalence of burn-out is an essential step for preventing workers from developing burn-out symptoms. Future research should work towards a holistic systems model on burn-out, including factors on the individual, organizational, and societal level. In addition, it is of major relevance to identify leverage points and key actions to intervene in the system and ultimately reduce the prevalence of burn-out.

## Data availability statement

The original contributions presented in the study are included in the article/Supplementary material, further inquiries can be directed to the corresponding author.

## Ethics statement

The studies involving humans were approved by Centre for Clinical Expertise at the RIVM. The studies were conducted in accordance with the local legislation and institutional requirements. The participants provided their written informed consent to participate in this study.

## Author contributions

LB: Formal analysis, Investigation, Methodology, Software, Visualization, Writing – original draft. SB: Conceptualization, Formal analysis, Investigation, Methodology, Supervision, Writing – review & editing. SL: Investigation, Writing – review & editing. MN: Writing – review & editing. SO: Conceptualization, Funding acquisition, Investigation, Methodology, Project administration, Supervision, Writing – review & editing.

## References

[1] Aumayr-Pintar Christine Cerf Catherine Parent-Thirion Agnès. (2018). “Burnout in the workplace: a review of the data and policy responses in the EU.”

[2] Hassard Juliet Teoh Kevin Cox Tom Cosmar M Gründler R Flemming D. (2014). “Calculating the cost of work-related stress and psychosocial risks.”

[3] Wolvetang S Maria J van Dongen E Speklé PC Schaafsma F. Sick leave due to stress, what are the costs for Dutch employers? J Occup Rehabil. (2022) 32:764–72. doi: 10.1007/s10926-022-10042-x, PMID: 35575823 PMC9109658

[4] Schaufeli WB. Burnout in Europe: relations with national economy, governance, and culture In: Research Unit Occupational & Organizational Psychology and professional learning (internal report). Belgium: KU Leuven (2018)

[5] van den Heuvel SG van Thor JAF Beiro L Ferandez van Dam L Driven HJ. (2022). “Nationale Enquete Arbeidsomstandigheden 2021 in vogelvlucht.”

[6] Koppes LLJ de Vroome EMM Mol MEM Janssen BJM van den Bossche SNJ. Nationale Enquête Arbeidsomstandigheden 2010: Methodologie en globale resultaten, [National Working Conditions Survey (NWCS) 2010: Methodology and overall results]. Hoofddorp, TNO (2011).

[7] World Health Organization. (2019). Burn-out an “occupational phenomenon”: International classification of diseases.

[8] Edu-Valsania S Laguia A Moriano JA. Burnout: a review of theory and measurement. Int J Environ Res Public Health. (2022) 19:1780. doi: 10.3390/ijerph19031780, PMID: 35162802 PMC8834764

[9] Schaufeli WB Salanova M González-Romá V Bakker AB. The measurement of engagement and burnout: a two sample confirmatory factor analytic approach. J Happiness Stud. (2002) 3:71–92. doi: 10.1023/A:1015630930326

[10] Vinkers CH Schaafsma FG. Burnout urgently needs robust research. Nature. (2021) 592:188–8. doi: 10.1038/d41586-021-00896-1, PMID: 33824518

[11] Salvagioni DAJ Melanda FN Mesas AE González AD Gabani FL Andrade SMD. Physical, psychological and occupational consequences of job burnout: a systematic review of prospective studies. PLoS One. (2017) 12:e0185781. doi: 10.1371/journal.pone.0185781, PMID: 28977041 PMC5627926

[12] Shoman Y El May E Marca SC Wild P Bianchi R Bugge MD. Predictors of occupational burnout: a systematic review. Int J Environ Res Public Health. (2021) 18:9188. doi: 10.3390/ijerph18179188, PMID: 34501782 PMC8430894

[13] Naczenski LM de Vries JD van Hooff MLM Kompier MAJ. Systematic review of the association between physical activity and burnout. J Occup Health. (2017) 59:477–94. doi: 10.1539/joh.17-0050-RA, PMID: 28993574 PMC5721270

[14] Söderström M Jeding K Ekstedt M Perski A Åkerstedt T. Insufficient sleep predicts clinical burnout. J Occup Health Psychol. (2012) 17:175–83. doi: 10.1037/a0027518, PMID: 22449013

[15] Verhavert Y De Martelaer K Van Hoof E Van Der Linden E Zinzen E Deliens T. The association between energy balance-related behavior and burn-out in adults: a systematic review. Nutrients. (2020) 12:397. doi: 10.3390/nu12020397, PMID: 32024269 PMC7071204

[16] Purvanova RK Muros JP. Gender differences in burnout: a meta-analysis. J Vocat Behav. (2010) 77:168–85. doi: 10.1016/j.jvb.2010.04.006

[17] Verhofstadt E De Witte H Omey E. Demand, control and its relationship with job mobility among young workers. Econ Ind Democr. (2009) 30:266–93. doi: 10.1177/0143831X09102434

[18] Bakker AB Demerouti E Sanz-Vergel A. Job demands-resources theory: ten years later. Annu Rev Organ Psychol Organ Behav. (2021) 10:25–53. doi: 10.1146/annurev-orgpsych-120920-053933

[19] Shin H Park YM Ying JY Kim B Noh H Lee SM. Relationships between coping strategies and burnout symptoms: a meta-analytic approach. Prof Psychol Res Pract. (2014) 45:44–56. doi: 10.1037/a0035220

[20] Brotheridge CM Grandey AA. Emotional labor and burnout: comparing two perspectives of “people work”. J Vocat Behav. (2002) 60:17–39. doi: 10.1006/jvbe.2001.1815

[21] Zapf D Seifert C Schmutte B Mertini H Holz M. Emotion work and job stressors and their effects on burnout. Psychol Health. (2001) 16:527–45. doi: 10.1080/08870440108405525 22804497

[22] Aybas M Elmas S Dündar G. Job insecurity and burnout: the moderating role of employability. Eur J Bus Manag. (2015) 7:195–203.

[23] Aronsson G Theorell T Grape T Hammarström A Hogstedt C Marteinsdottir I. A systematic review including meta-analysis of work environment and burnout symptoms. BMC Public Health. (2017) 17:1–13. doi: 10.1186/s12889-017-4153-7 28302088 PMC5356239

[24] Madsen IEH Nyberg ST Magnusson Hanson LL Ferrie JE Ahola K Lars Alfredsson G. Job strain as a risk factor for clinical depression: systematic review and meta-analysis with additional individual participant data. Psychol Med. (2017) 47:1342–56. doi: 10.1017/S003329171600355X, PMID: 28122650 PMC5471831

[25] Dollard MF Opie T Lenthall S Wakerman J Knight S Dunn S. Psychosocial safety climate as an antecedent of work characteristics and psychological strain: a multilevel model. Work Stress. (2012) 26:385–404. doi: 10.1080/02678373.2012.734154

[26] Anttila T Härmä M Oinas T. Working hours–tracking the current and future trends. Ind Health. (2021) 59:285–92. doi: 10.2486/indhealth.2021-0086, PMID: 34421102 PMC8516630

[27] Howard J. Algorithms and the future of work. Am J Ind Med. (2022) 65:943–52. doi: 10.1002/ajim.23429 36128686

[28] Thoits PA. Stress and health: major findings and policy implications. J Health Soc Behav. (2010) 51:S41–53. doi: 10.1177/0022146510383499, PMID: 20943582

[29] Turner JR Baker RM. Complexity theory: an overview with potential applications for the social sciences. Systems. (2019) 7:4. doi: 10.3390/systems7010004

[30] Veldhuis GA Sluijs T van Zwieten MHJ Bouwman J Wiezer NM Wortelboer HM. A proof-of-concept system dynamics simulation model of the development of burnout and recovery using retrospective case data. Int J Environ Res Public Health. (2020) 17:5964. doi: 10.3390/ijerph17165964, PMID: 32824546 PMC7459661

[31] Hovmand PS. Group model building and community-based system dynamics process. New York: Springer (2014).

[32] Andersen DF Vennix JAM Richardson GP Rouwette EAJA. Group model building: problem structuring, policy simulation and decision support. J Oper Res Soc. (2007) 58:691–4. doi: 10.1057/palgrave.jors.2602339

[33] Hovmand PS Andersen DF Rouwette E Richardson GP Rux K Calhoun A. Group model-building ‘scripts’ as a collaborative planning tool. Syst Res Behav Sci. (2012) 29:179–93. doi: 10.1002/sres.2105

[34] Sterman J. Business dynamics: System thinking and modeling for a complex world. Irwin McGraw-Hill: Boston, MA, USA (2001).

[35] Maani K. Systems thinking. World Scientific Book Chapters. (2017):15–23.

[36] Meadows DH. Thinking in systems: A primer. Vermont, USA: Chelsea green publishing (2008).

[37] National Institute for Public Health and the Environment. (2021). Impactvolle Determinanten: Psychosociale arbeidsbelasting Available at: https://www.rivm.nl/sites/default/files/2021-07/LR_012065_131709_Factsheet%20_arbeidsbelasting_V4.pdf

[38] Dahlgren G Whitehead M. The Dahlgren-Whitehead model of health determinants: 30 years on and still chasing rainbows. Public Health. (2021) 199:20–4. doi: 10.1016/j.puhe.2021.08.009, PMID: 34534885

[39] Niks IMW Veldhuis GA van Zwieten MHJ Sluijs T Wiezer NM Wortelboer HM. Individual workplace well-being captured into a literature-and stakeholders-based causal loop diagram. Int J Environ Res Public Health. (2022) 19:8925. doi: 10.3390/ijerph19158925, PMID: 35897299 PMC9331132

[40] Baugh Littlejohns L Hill C Neudorf C. Diverse approaches to creating and using causal loop diagrams in public Health Research: recommendations from a scoping review. Public Health Rev. (2021) 42:1604352. doi: 10.3389/phrs.2021.1604352 35140995 PMC8712315

[41] Schaufeli WB Bakker AB. Job demands, job resources, and their relationship with burnout and engagement: a multi-sample study. J Organ Behav. (2004) 25:293–315. doi: 10.1002/job.248

[42] Jeon Y-H Luscombe G Chenoweth L Stein-Parbury J Brodaty H King M. Staff outcomes from the caring for aged dementia care resident study (CADRES): a cluster randomised trial. Int J Nurs Stud. (2012) 49:508–18. doi: 10.1016/j.ijnurstu.2011.10.020, PMID: 22078076

[43] Fritz C Ellis AM Demsky CA Lin BC Guros F. Embracing work breaks. Organ Dyn. (2013) 42:274–80. doi: 10.1016/j.orgdyn.2013.07.005

[44] Meijman TF Mulder G. Psychological aspects of workload In: A handbook of work and organizational psychology. UK: Erlbaum (2013). 15–44.

[45] Scott RJ Cavana RY Cameron D. Recent evidence on the effectiveness of group model building. Eur J Oper Res. (2016) 249:908–18. doi: 10.1016/j.ejor.2015.06.078

[46] Aust B Møller JL Nordentoft M Frydendall KB Bengtsen E Jensen AB. How effective are organizational-level interventions in improving the psychosocial work environment, health, and retention of workers? A systematic overview of systematic reviews. Scand J Work Environ Health. (2023) 49:315–29. doi: 10.5271/sjweh.4097, PMID: 37158211 PMC10713994

