# Supplementary material for: A system science perspective on burn-out: development of an expert-based causal loop diagram
Source: Front Public Health. 2023 Nov 16;11:1271591. doi: 10.3389/fpubh.2023.1271591 (PMC10687398; doi:10.3389/fpubh.2023.1271591)
Supplement: Supplementary file 1 [file Table_1.DOCX]

Supplementary Material

# Supplementary table 1. Overview of the iterative process of building the CLD.

| **Factors^a^** | **Workshop 1^b^** | **In between** | **Workshop 2** | **In between** | **Following Workshop 3** |
| --- | --- | --- | --- | --- | --- |
| Overview of determinants of burn-out |  |  |  |  |  |
| *Living conditions* |  |  |  |  |  |
| **Financial stress** | 3 stickers |  |  |  |  |
| Poor housing | 2 stickers |  |  |  |  |
| **Good social network** | 3 stickers | Renamed: Social network |  |  |  |
|  |  |  |  |  |  |
| *Working conditions* |  |  |  |  |  |
| **Emotionally demanding work situations** | 4 stickers |  |  |  |  |
| **Aggressive behavior at work** | 2 stickers |  | Listed for selection; **selected** |  |  |
| **Low supervisor support** | 5 stickers  Renamed: Functional supervisor support |  |  |  |  |
| **Low coworker support** | 0 stickers^c^ |  | Listed for selection; **selected**  Renamed: Co-worker support |  |  |
| **Low autonomy** | 0 stickers |  | Listed for selection; **selected**  Renamed: Autonomy |  |  |
| **Having to work (extra) hard** | 3 stickers | Renamed: (Very) high workload |  |  |  |
| Mismatch between knowledge/skills and job | 1 sticker |  |  |  |  |
|  |  |  |  |  |  |
| *Societal developments* |  |  |  |  |  |
| Need for new skills | 3 stickers |  | Listed for selection |  |  |
| 24/7 economy | 0 stickers |  |  |  |  |
| Uncertainty | 1 sticker |  |  |  |  |
| **Small working population** | 4 stickers | Renamed: Size of working population |  |  |  |
| Shortages at the labor market | 1 sticker |  |  |  |  |
| **Occupational health and safety (OHS) legislation** |  |  | Listed for selection; **selected** |  |  |
| Combining tasks (caregiving, work, volunteering, private, etc.) | 0 stickers |  |  |  |  |
| **Disturbed work-life balance** | 4 stickers |  |  |  | Divided in two:  Work interference with private life, private life interference with work |
|  |  |  |  |  |  |
| Additional factors |  |  |  |  |  |
| Effort reward imbalance | 2 stickers |  |  |  |  |
| **Task clarity** | 2 stickers |  | Listed for selection; **selected** |  |  |
| Temporary contracts | 1 sticker |  |  |  |  |
| Safe organizational culture | 0 stickers |  |  |  |  |
| Hierarchical organizational culture | 0 stickers |  |  |  |  |
| **Limited access to mental health care** | 2 stickers |  | Listed for selection; **selected** |  |  |
| **Rising costs of living** | 4 stickers | Renamed: Costs of living |  |  |  |
| **Rougher social climate** | 3 stickers |  | Listed for selection; **selected** |  |  |
| Curling parenting | 3 stickers |  | Listed for selection; **selected** |  | Removed from CLD |
| Consequences of the two-earner model | 1 sticker |  |  |  |  |
| Market competition in public sectors | 0 stickers |  |  |  |  |
| **Worsening social security** | 3 stickers |  | Listed for selection; **selected** |  |  |
| **Job security** |  |  | Listed for selection; **selected** |  |  |
| Well-functioning works/participation |  |  | Added by participants; **selected** | Removed from CLD |  |
| Learning and development opportunities |  |  | Added by participants; **selected** | Removed from CLD |  |
| **Recovery time** |  |  | Added by participants; **selected** |  |  |
| Work pressure |  |  | Added by participants; **selected** | Removed from CLD |  |
| **Compliance with occupational health and safety legislation** |  |  | Added by participants;  **selected** | Renamed: Healthy work(place) culture |  |

*Note*: Factors from the overview of determinants of burn-out that were not prioritized: working conditions: emotionally involved in work, physical violence, labor dispute, stressful events, risk of accidents, bullying, discrimination, (sexual) intimidation, type of work (sector), position within organization, resumption of work, isolated work, low social support at the workplace, future uncertainty, salary: piece-wages/piecework, high job demands, lack of control, unfavorable organizational structure, inadequate organizational management, inadequate organizational culture; societal developments: robotization, having to work faster, night work, flexible contracts, flexibilization of the labor market, platform economy, migration, Risk Assessment and Evaluation (RAE), employers’ policy on psychosocial working conditions, Netherlands Labor Authority

^a^Bold factors were selected for the CLD.

^b^Each of the 7 experts received 10 stickers (5 for living and working conditions and 5 for societal developments); several stickers could be placed on one factor; in total 70 stickers were distributed.

^c^Factors with 0 stickers were selected by the experts but not prioritized.
